# Frailty Focus: Empowering Rural Health With Advanced Nurse Practitioners: A Discussion Paper

**DOI:** 10.1111/jep.70075

**Published:** 2025-04-01

**Authors:** Maria Betts, Deirdre Harkin

**Affiliations:** ^1^ Institute of Nursing and Health Research Ulster University Belfast Northern Ireland UK; ^2^ School of Nursing and Paramedic Sciences Ulster University Belfast Northern Ireland UK

**Keywords:** advanced nurse practitioner, frailty, older person, public health, rurality

## Abstract

**Background:**

Frailty is a multidimensional condition with symptoms relating to falls, immobility, incontinence, impaired memory and medication side‐effects. With increasing numbers of frailty, particularly in rural areas, healthcare systems are being challenged globally. Moreover, frailty may be more common in rural communities as a consequence of transportation issues, limited access to healthcare services and health promotion activities. Advanced Nurse Practitioners are ideally placed to undertake comprehensive geriatric assessments and identify frailly syndromes.

**Aims:**

Explore the function of the Advanced Nurse Practitioner in managing people living with frailty in rural areas, drawing on a review of current guidelines, literature, and practice, considering public health agendas and evidence‐based practice.

**Design:**

Discussion paper.

**Key Learnings:**

Frailty is linked to poorer health outcomes, an excessive use of health resources and decreased quality of life. Incorporating Advanced Nurse Practitioners into integrated models of care and health improvement strategies, can improve patient outcomes.

**Conclusion:**

Using advanced clinical and decision‐making skills, Advanced Nurse practitioners deliver evidence‐based care to improve patient safety and health outcomes. Creating partnerships to enhance the provision of healthcare, they are focused on frailty prevention, detection and providing support to develop co‐produced management plans to address individual needs. The Advanced Nurse Practitioner has the ability to practice autonomously within an expanded scope of clinical practice, making them the ideal professional to support people living with frailty in rural areas.

**Impact for Nursing:**

When thinking about future strategies for advanced practice, it is important to acknowledge the lack of regulation, roles in nursing, inadequate title protection, role variability, and different educational requirements. Organisations need to consider the enablers and barriers of Advanced Nurse Practitioners fulfilling their duties. Advanced Nurse practitioners are guided by public health agendas to improve the population health of those in rural areas.

**No Patient or Public Contribution:**

This is a discussion paper.

## 1 Introduction

Modern healthcare can extend life, though the accompanying ramifications for the older person may ultimately involve an increased risk of certain health conditions such as frailty and can have unintentional effects on patient safety [1]. Frailty is a chronic condition associated with ageing where many body systems deplete their natural reserves (British Geriatric Society [BGS], 2022). Frailty is a condition and not a stereotype, it should be accompanied with a diagnosis made by a healthcare professional (BGS, 2022). The prevalence of frailty among older people living in communities varies globally, with Siriwardhana et al., [2] noting that frailty is more common among community‐dwelling older adults in middle‐income countries such as Cuba than in wealthier countries such as China, highlighting significant considerations for healthcare planning. Limited research has examined frailty in low‐income countries, though Gray et al., [3] found that older people living in rural Tanzania were more likely to be living with frailty than those in middle or high‐income countries. Nonetheless, the consistent innovations over the last twenty years have contributed to the increased recognition of frailty by geriatricians as well as subspecialists like cardiologists and advanced practitioners, for whom making decisions regarding older persons is a daily occurrence [4].

Frail older adults are more likely to be hospitalised and experience complications from hospital admissions, such as infection, delirium, and falls [5]. Integrating frailty assessment into current or new care models may help identify the most vulnerable patients who could benefit from effect care transitions. The National Health Service in the United Kingdom (UK) is a free service to service users with shared care models a priority [6]. Community care is delivered by a range of healthcare professionals such as carers, nurses, occupational therapists, physiotherapist, medics and Advanced Nurse practitioners (ANP). Patient safety is a high priority within the frail population, particularly in rural areas where patients are often overlooked [1]. This discussion article will set the scene for the function of the ANP in managing people living with frailty in rural areas, considering public health agendas, evidence‐based practice and patient safety. The intended audience of the paper is all community healthcare professionals who deliver care to service users. It also aims elicit the attention of healthcare professionals working in the hospital and voluntary sectors and highlighting the importance of the topic to organisations and policymakers. The paper will discuss how policy and public health agendas direct the care and resources for this client group and how the ANP can play a pivotal role in population health and safety.

## 2 Background

Frailty affects an estimated 10% of adults over 65 years and 50% over 85 years in the UK [7] and continues to challenge healthcare systems globally [8]. Advancing age, greater levels of deprivation, female gender, Asian ethnicity, and living in urban areas have been linked to increase the prevalence of frailty progressing more rapidly, resulting in a higher level of prevalence [9]. The ageing population may be socially isolated, lonely, and live with a plethora of health condition, which can result in the additional diagnosis of frailty [9]. The prevalence of older communities and subsequent frailty is increasing exponentially in the UK, particularly in rural areas [10]. Frailty has been reported to be more common in rural communities with reduced access to healthcare services and health promotion activities [11]. Rural health is the health of people living in rural areas, who generally are located farther from health care facilities and other services than people living in urban areas [12].

Frailty is a multidimensional condition with a compilation of factors relating to falls, immobility, incontinence, impaired memory, and medication side‐effects [8]. This reduces a person's resilience, increases vulnerability to loss of independence and risk of hospital admissions (Hill and Fox, 2023). The presence of frailty is linked to poorer health outcomes, an increased use of health resources and decreased quality of life when compared to the non‐frail population [5]. There is a dearth of research examining older people's experience of living with frailty, possibly due to exclusion criteria based on multimorbidity's and age [1]. Rectifying this issue would have immeasurable benefits; therefore, an acceleration into researching frail participants is required to fill the gaps in the evidence‐base if frailty is to be addressed (Table 1 for elements of frailty).

**Table 1:** Elements of frailty.

In the older person
Distinctive health state related to the ageing process
In the person with chronic illnesses—not always frailty but can be a prerequisite
Frailty vs frailty syndromes
The frailty state can fluctuate depending on contributing factors

*Note:* British Geriatric Society [13].

Frailty and global deterioration associated with aging are both concepts that describe declines in health and functioning, but they differ in scope, underlying mechanisms, and manifestations. Frailty is a specific clinical syndrome characterised by increased vulnerability to stressors due to reduced physiological reserves and dysregulation of multiple systems with physical, cognitive, and social components (BGS 2019). Global deterioration with aging refers to the general decline in physical, cognitive, and functional abilities that occurs naturally with aging [14]. It is not a specific syndrome but an umbrella term describing various age‐related changes. Therefore, as frailty is driven by systemic changes such as chronic inflammation, loss of muscle mass, and malnutrition, it reflects an accelerated decline beyond what is typical for aging, highlighting the need for accurate diagnosis and treatment of frailty.

In Ireland, people living with frailty account for 22% of emergency department (ED) attendances [15], highlighting the need to create clinical services that go beyond the conventional ways of providing care to guarantee that patients receive coordinated care and support. Hough [16] demonstrated that while developing their role to meet the demands and changes in the workforce, ANPs increased patient access to services. A qualitative research study completed by Woo et al. [17] examined patients' perceptions of the ANP role in Singapore with results indicating that incorporating ANPs into integrated models of care improved patient satisfaction and health outcomes. In the UK, the implementation of the ANP role in district nursing has been gathering pace in the past 5 years with government policy support [18, 19].

## 3 Discussion

### 3.1 The Advanced Nurse Practitioner Role

Nurses play a critical role to address patient safety issues, to develop, implement and evaluate solutions to improve quality of care and reduce risks of harm. Moreover, according to the International Council of Nurses (ICN) in 2020, the role of the nurse practitioner is influenced by the environment in which they are approved to practice. The ANP responds to the increasing needs of people with frailty by working within an extended scope of practice using a person‐centred approach [18]. Utilising highly evolved diagnostic, assessment, analytical and clinical judgment skills promotes health improvement, allowing the patient to age well [18]. Within the community, ANPs have become an integral part of the multidisciplinary team (MDT), assessing and treating older people living with frailty in their own homes in an aim to promote independence and help people to remain in their own homes.

Frailty has been predominantly managed by care of the older person consultant‐led clinics; though a shortage of geriatricians and the willingness to work in rural areas has resulted in uncoordinated care and management (Irimia et al., 2021). An alternative method of care delivery is required to address this impending crisis for both patients and healthcare organisations. The National Health Service's (NHS) Long‐Term Plan [20] aims to reduce the volume of older people being admitted to hospital and shorten their length of stay by providing easier access to community healthcare professionals. Advanced Nurse Practitioners strive to reduce uncoordinated care and ED attendances through health improvement strategies among the frail community [21].

Advanced Nurse Practitioners are in a unique position to use their skills and experience to deliver evidence‐based care and improve health. Ryley and Middleton [22] report that the ANP ought to be considered as a level of practice as opposed to a role. Studies have found that patients are unaware of the higher level of education and training that an ANP undertakes [23]. Research by Htay and Whitehead [24] has proven that the ANP's role results in patient satisfaction, improved patient outcomes, control of chronic disease, and cost‐effectiveness.

Advanced Nurse practitioners create partnerships to enhance the provision of healthcare, as they are focused on frailty prevention, detection, and providing individuals with appropriate support according to their needs [19, 25]. Using advanced diagnostic, analytical, critical thinking, and clinical assessment skills, the ANP co‐produces management plans with patients and their families [21]. The ANP's ability to practice autonomously within an expanded scope of clinical practice [18] makes them the ideal professional to manage and support people living with frailty in rural areas. ANPs practice in compliance with the Nursing and Midwifery Council (NMC) Code [26] and are additionally guided by the DHSSPS [18] four pillars of advanced practice, including direct clinical practice, supported by education and learning, research, and evidence‐based practice and leadership and collaborative practice. Thus, this compliance provides a structure to the role that benefits patients, healthcare professionals, and healthcare organisations.

### 3.2 Risk Stratification/Frailty Assessment Tools

Scholes‐Robertson et al., [27] demonstrated that people living in rural areas experience loneliness, depression, isolation, and reduced self‐esteem with lacking access to timely and appropriate care resulting in suboptimal health outcomes. Risk factors for frailty include ageing, cognitive decline, depression, poor diet, difficulty performing activities of daily living, and low self‐awareness of health [28]. England's National Health Service is the first healthcare system globally to systematically identify individuals aged 65 and older living with moderate to severe frailty through a population‐based stratification method. This process can be carried out using the electronic Frailty Index (eFI) or other suitable assessment tools. The eFI leverages routine health record data to automatically generate a score, indicating whether a person is likely fit or experiencing mild, moderate, or severe frailty [29]. Recognising critical frailty markers help identify those at risk, prompting the ANP to use risk stratification screening tools, such as the Rockwood Clinical Frailty Score (CFS) [30], optimising healthcare by slowing progression and signposting patients to the right services [31]. Other tools which aim to increase patient safety include Fragility Fracture Calculations (FRAX), Anticholinergic Burden Score (ACB), and Falls Risk Assessments. These tools are aimed at identifying risk factors that may be present for older people and how the ANP can address these with medications optimisation by stopping drugs that can increase the risk of falls, delirium and polypharmacy. Though reducing polypharmacy is important, it may be appropriate to prescribe medications such as bisphosphonates to improve bone health. The overall health of the patient must be taken into consideration. The ability of the ANP to integrate various components of a complicated patient picture through clinical skill, leadership, and collaborative practice enhances patient safety and outcomes [32].

Patient safety for frail older people living in rural areas is particularly challenging due to reduced access to clinical and social services for those who need it most, such as the frail population [33]. With evidence of reduced access to healthcare services [11], telehealth/telemedicine has played a crucial role in connecting patients with healthcare professionals. This enables doctors and nurses to use electronic information and communications technologies to provide support to patients living with frailty in rural areas and create treatment plans or give health promotion advice as assessed [34]. This was particularly evident during the coronavirus pandemic where patients in rural areas were less likely to have face‐to‐face interactions with healthcare staff (Driscoll and Cartledge, 2022). The Advanced Nurse Practitioner can use telemedicine to reduce the risk of the patient having a health event that may otherwise go undetected. This information is shared among professionals but a responsible person should be identified as the coordinator, with enhanced clinical and leadership skills, the Advanced Nurse Practitioner ideally placed to take on this role.

### 3.3 Collaborative Practice

The ANP uses collaborative practice to coordinate services, assuming greater responsibility, in terms of both nursing and medical care (Ljungbeck et al., 2017). A lack of collaborative working has been found to negatively impact patient safety with a qualitative research study by Sheard et al., (2017) highlighting senior positions, cultures, and leadership as barriers. Public health agendas such as the NHS Right‐Care; Frailty Toolkit (2019) and the Enhancing Clinical Care Framework [35], can improve population health. A study by Kuehnert et al. [36] supports these agendas with policy‐driven, population‐focused nursing actions resulting in improved quality of life for older people.

The ANP takes the lead in population health, forming alliances with various stakeholders. They employ methods to enhance wellbeing and health such as comprehensive geriatric assessments (CGA); a multifaceted, diagnostic assessment which gathers data on an elderly person's physical, mental, and functional capabilities (BGS, 2019). Historically completed by geriatricians, CGAs help identify those at risk of frailty and tailor care to their individual needs to prevent further deterioration and improve quality of life (BGS, 2022). It has further developed as a multi‐professional approach ensuring an understanding of the older person's health. Though, McCann et al. [37] found that it lacked thorough assessment in areas of pain, depression, and advanced care planning. However, interventions based on a CGA can decrease mortality and increase functional capacity in older people [38].

The ANP is seen as a hybrid between the nurse and doctor roles, as they combine the duties of a nurse in providing care with the ability to make medical treatment decisions [31]. Though, Mannix and Jones [39] found that ANPs became trapped between nursing and medicine, fearing an identity crisis. Patients appreciate the holistic approach by the ANP, bridging the mistakes and delays that can occur as a result of disjointed healthcare systems [40].

### 3.4 Comprehensive Geriatric Assessment (CGA)

A CGA is one of many assessments used to assess older people. A CGA is a process of care comprising of. Number of steps and is the assessment of choice in the United Kingdom due to its multidimensional holistic assessment considering health and wellbeing [41]. Completing a CGA at home can enable better awareness of social and practical situations as these may differ when a patient is in hospital. Chen et al., [38] found that CGAs reduce caregiver burden and improve independence when compared to usual care. The ANP has been recognised as the ideal professional to complete a CGA, identify frailty syndromes and develop management plans to address individualised needs [42]. Frailty must be a diagnosis, with the British Geriatric Society guidelines (2019) recommending that the five frailty syndromes of falls, immobility, delirium, incontinence, and the side‐effects of medications can be used to aid this. Shared‐decision‐making and coproduction of care plans engages the patient to partake in interventions that may improve their physical ability and cognitive function [43]. Promoting health through fall prevention strategies, cognitive stimulation, chronic disease management, advance care planning, patient education, and regular follow‐ups are part of the ANP's role [32]. Incorporating falls risk assessment tools and fragility fracture calculators help identify those most at risk of frailty, enabling preventative measures to be instigated [44, 48].

### 3.5 Four Pillars of Advanced Practice

The DHSSPS [18] framework for Advanced Nursing Practice was introduced to facilitate a methodical and coherent approach to the creation and execution of the Advanced Nurse Practitioner role in Northern Ireland. A significant amount of research shows the benefits of advanced nurse practitioners contributing to different healthcare settings [46, 47]. Research indicates that ANP positions have expanded globally and changed within several specialisation fields to become extremely versatile and productive [48]. These studies demonstrate how advanced nurses improve health outcomes, and they have also added to the body of evidence supporting advanced nursing practice. The ANP is able to guide their role using the four pillars of advanced practice to deliver effective clinical care to the population whilst using their leadership and research skills to deliver evidence‐based practice.

#### 3.5.1 Direct Clinical Practice

The ANP uses clinical reasoning to gather and analyse patient data, problem‐solve, establish differential diagnosis, and direct treatment using specialised knowledge [45]. However, the ANP's knowledge, experiences and self‐awareness can impact clinical reasoning. To promote shared decision‐making during treatment planning, raise patient satisfaction levels, and promote independence, effective communication is essential [45].

A literature review by Thompson et al. [49] has shown that ANP consultations were longer with patients than doctors, with comparable diagnosis and therapeutic treatments. This may be owing to the ANP's Master's level education, which focuses on person‐centred and evidence‐based approaches [40]. Effective history‐taking, physical examination, person‐centredness, and active listening, form the basis for clinical reasoning and differential diagnosis [49]. Stoop et al. [50] suggest that a key factor in patient dissatisfaction is the absence of their involvement in the CGA. The ANP should ensure patients understand any investigations and managements plans that are recommended [48].

Medication reviews are a key element of the CGA, with the ANP well‐placed to manage patients with frailty and polypharmacy living in rural areas as they can provide an entire episode of care [51]. Patients with frailty are more susceptible to medication side‐effects and often take Falls Risk Increasing Drugs (FRIDS) and medications with Anticholinergic Burden (ACB) effects, which can cause falls, confusion, delirium and hallucinations [52]. ANPs can reduce inappropriate polypharmacy, ACB and FRIDs scores as potential health promotion and improvement [52]. Involving patients in the process of making decisions is essential, including management plans to promote concordance not least in care homes where residents, relatives and staff are involved [51, 53]. ANPs utilise their leadership skills in care homes to assist staff to promote wellbeing [35].

#### 3.5.2 Leadership and Collaborative Practice

ANPs assume a variety of leadership responsibilities given that they practice autonomously within their advanced scope of practice (DH, 2016 [54]). Though Lamb et al. [55] recognise it as a lesser‐known aspect of the ANP role, even by ANPs themselves. ANPs are firmly positioned as leaders due to their innovative approaches to providing alternative care and education for those with frailty [54]. Equipping patients, family and carers with the knowledge to be proactive in health‐management may positively impact how health promotion and self‐management can determine population health [56]. The principles of integrated care and collaborative working are part of the ANP role and one which is crucial in the delivery of a safe service for the frail population [50].

#### 3.5.3 Education and Learning

Advanced Nurse Practitioners lead educational MDT meetings to enhance the knowledge of other healthcare professionals and ensure care delivered is evidence‐based (Dias Torres [57]). Person‐centred leadership practice is substantially correlated with leaders' experiences of cooperation, coordination, communication, concerns, values, and beliefs, creating trustworthy attitudes and fostering strong relationships [58]. Outcomes for patients and staff are enhanced by this positive culture, encouraging creativity and lessening the need for bureaucracy and control [58]. This leadership style allows the ANP to work collaboratively with the multi‐professional frailty team as set out in the ANP Framework [18].

#### 3.5.4 Research and Evidenced‐Based Practice

ANPs require research skills and critical analysis to conduct scientific inquiry to advance the profession [59]. The ANP interprets research findings to deliver evidenced‐based care, that is, Rockwood Clinical Frailty Tool. They have a responsibility to monitor and enhance the standard of treatment and practice efficacy [18]. Furthermore, McConkey et al. [59] recommend a hybrid approach to research and evidence‐based practice with clinicians and academics working in tandem.

## 4 Implications for Nursing and Rural Communities

When thinking about future strategies for the ANP function and guaranteeing patient safety, it is important to consider the familiar problems with the absence of ANP regulation, inadequate title protection, role variability, and different educational requirements [60]. Organisations need to consider the enablers and barriers of the ANP fulfilling their duties to ensure effectiveness of the role, promoting patient safety, enhancing patient outcomes and experiences.

Growing evidence indicates that, to effectively integrate ANPs into the healthcare workforce, the healthcare environment must be considered, along with the healthcare demands of its population [61]. With the UK experiencing a swift rise in its aging population, the number of older people living with frailty in rural areas is poised to escalate. Hence, it becomes imperative to support this population group in maintaining safety and autonomy within their communities for as long as possible. Research has shown that older people living in rural areas experience lower socioeconomic status than those in urban areas (Huang et al. 2021), making the ANP role even more crucial to limit health inequalities and fair access to good healthcare. Implementation of the ANP role in rural communities can enhance the patient experience through nurse‐led multidimensional interventions which prove beneficial for this client group to support and promote health and independence. A literature review by Thompson et al. [49] highlighted that ANPs are key to the modernisation of the NHS. This is reflective of the approach that policymakers are taking to include the expertise of the nurse using advanced practice. The delivery of rapid and expert care in people's own homes is crucial if we are to promote health education and independence for those living with frailty in rural communities and ultimately improve health equity and outcomes.

## 5 Conclusion

Drawing on review of current guidelines, literature and practice within this paper, it is noted that an ageing population, with multifactorial health conditions such as frailty, has put pressure on an already pressurised healthcare system. When providing care for individuals with undifferentiated and undiagnosed needs, the ANP is responsible for the complete episode of care, using their complex decision‐making abilities and specialist knowledge [18]. This makes the ANP the most appropriate healthcare professional to undertake CGAs and provide seamless care for the patient. They are well placed to contribute to the transformation of health and care services. Guided by public health agendas to improve population health, the ANP provides expert advice to encourage self‐management. Playing a crucial role within the MDT, through coordinating care, educating staff, and completing research, the ANP role continues to thrive and receive respect. Regulation of the ANP role is required to further cement the benefits for patients and their families and optimise patient safety. Better understanding of the prevalence and effect of frailty on older people in rural populations will contribute to the development and implementation of policies and health interventions to deal with the emergence and advancement of frailty.

## Author Contributions

Maria Betts and Deirdre Harkin**:** Made substantial contributions to conception and design, or acquisition of data, or analysis and interpretation of data; Maria Betts and Deirdre Harkin**:** Involved in drafting the manuscript or revising it critically for important intellectual content; Maria Betts and Deirdre Harkin**:** Given final approval of the version to be published. Each author should have participated sufficiently in the work to take public responsibility for appropriate portions of the content; Maria Betts and Deirdre Harkin**:** Agreed to be accountable for all aspects of the work in ensuring that questions related to the accuracy or integrity of any part of the work are appropriately investigated and resolved.

## Conflicts of Interest

The authors declare no conflicts of interest.

## Data Availability

The authors have nothing to report.
